# Eosinophilic mesenteric vasculitis presenting as inflammatory bowel disease

**DOI:** 10.1002/jpr3.70035

**Published:** 2025-05-20

**Authors:** Razan Alkhouri, Craig Wong, Allyson Richards, David Martin, Joshua Hanson, Rasha Elmaoued, Rajmohan Dharmaraj, Ioannis Kalampokis

**Affiliations:** ^1^ University of New Mexico Albuquerque New Mexico USA; ^2^ University of Nebraska Medical Center Omaha Nebraska USA

**Keywords:** crohn's disease, eosinophilic vasculitis, inflammatory bowel disease

## Abstract

Inflammatory bowel disease (IBD), including Crohn's Disease (CD) and ulcerative colitis, is a chronic inflammatory condition affecting the gastrointestinal tract. Treatment for IBD depends on disease severity and can include medical and surgical management. Advances in treatment and the availability of biologics have significantly reduced the need for surgical interventions. Eosinophilic mesenteric vasculitis (EMV) is a rare form of intestinal vasculitis that can mimic IBD. Diagnosis of EMV is challenging as it requires full‐thickness biopsies. It can be mistaken for CD due to its response to steroids, which are a first‐line therapy for EMV; however, EMV typically does not respond to other IBD‐specific therapies. We present the case of a 15‐year‐old girl with a history of autoimmune hemolytic anemia who initially appeared to have CD but was diagnosed with EMV following a lack of clinical remission and persistence of the colonic stricture despite biologic therapy, which ultimately led to bowel obstruction symptoms requiring surgical resection.

## 1 INTRODUCTION

Inflammatory bowel disease (IBD), including Crohn's disease (CD) and ulcerative colitis (UC), is a chronic inflammatory condition affecting the gastrointestinal tract, with over 1.2 million individuals affected in North America.^1^ Common symptoms include abdominal pain, gastrointestinal bleeding, diarrhea, weight loss, and growth failure. Diagnosis is based on a combination of clinical, histological, biochemical, and imaging features.^2^ Treatment varies by severity and includes medical and surgical options. With the early utilization of biologics, the need of surgical intervention has been declining.^3^

Eosinophilic mesenteric vasculitis (EMV), a rare form of intestinal vasculitis, can present similarly to IBD. Diagnosing EMV is challenging as it requires full‐thickness biopsies. EMV often mimics CD due to a similar response to steroids. Additionally, the clinical presentations of EMV and CD, including symptoms such as abdominal pain, weight loss, and gastrointestinal bleeding, can overlap. Both conditions may show elevated inflammatory markers, strictures on imaging, and extraintestinal manifestations, further complicating differentiation.^4^ This case emphasizes the need to differentiate between IBD and EMV in patients with refractory symptoms.

## 2 CASE REPORT

**Figure 1:**
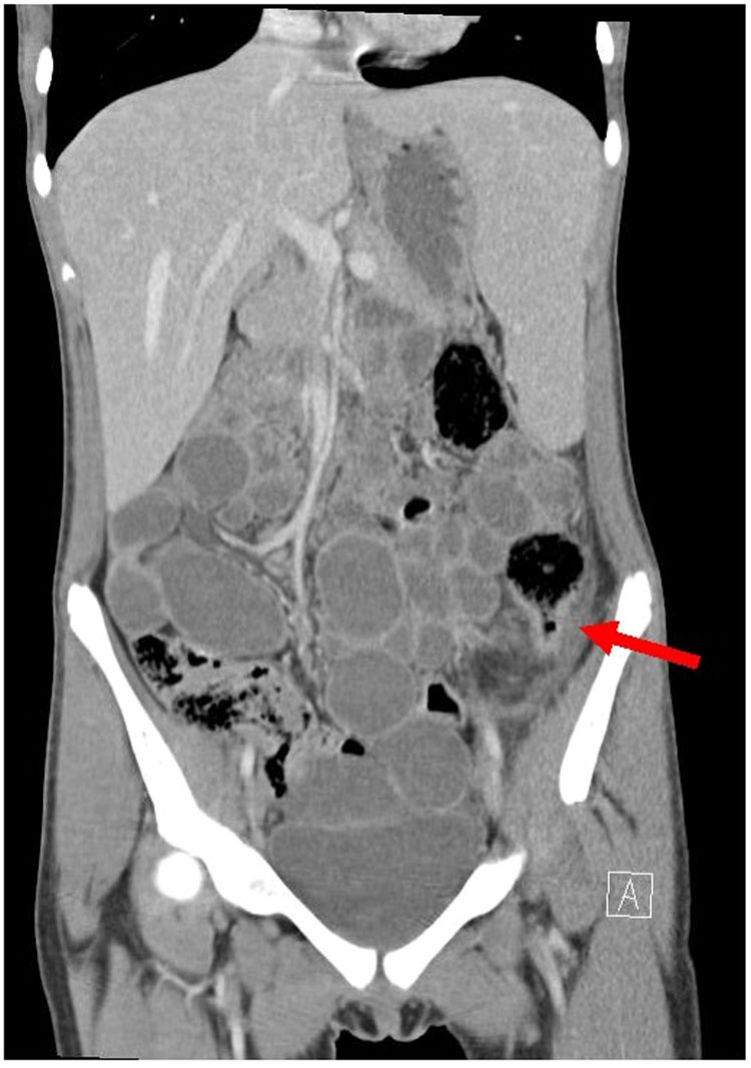
Computed tomography abdomen and pelvis with IV contrast. Coronal image showing abnormal narrowing at the descending colon as denoted by the arrow.

**Figure 2:**
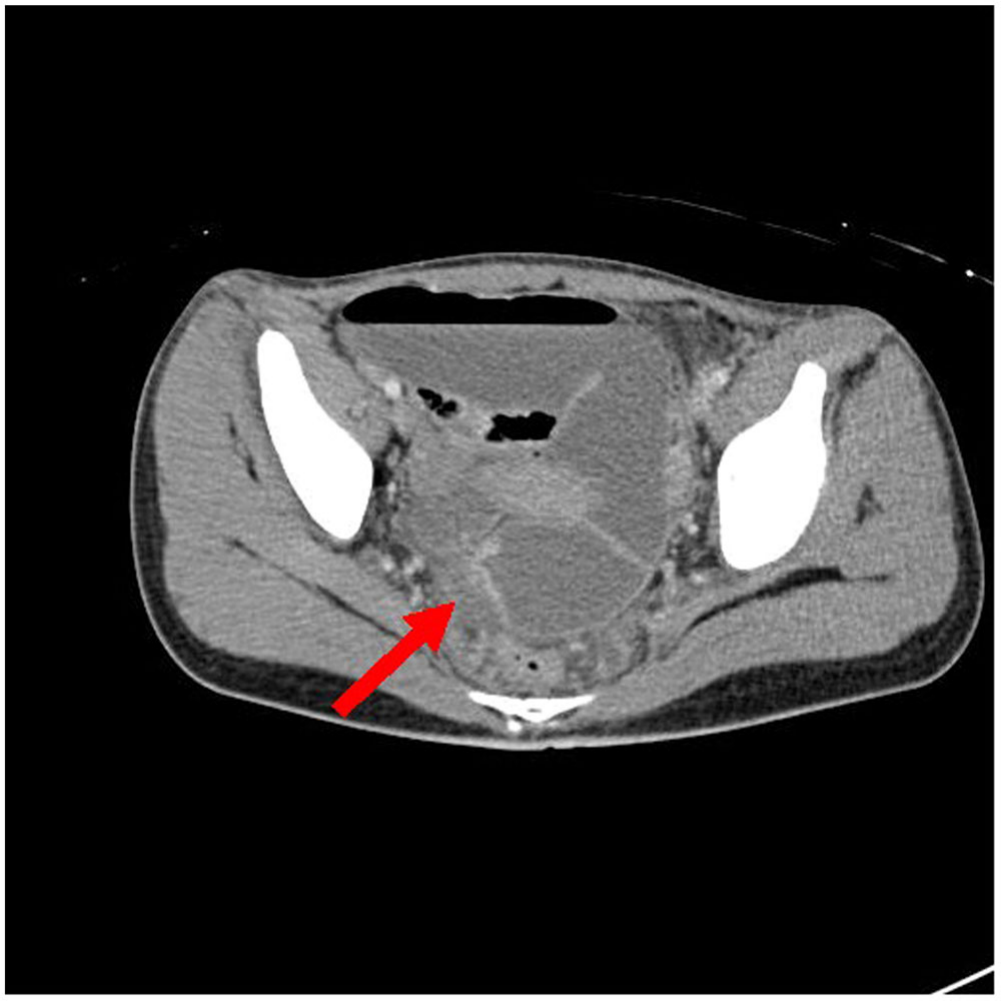
Computed tomography abdomen and pelvis with IV contrast. Axial image showing the distal ileal stricture as denoted by the arrow.

A 15‐year‐old adopted Chinese female admitted for autoimmune hemolytic anemia workup, was found to have 2 months of intermittent lower abdominal pain, 15‐pound weight loss, vomiting, and joint pain. She had no diarrhea or hematochezia. Physical examination revealed symmetric polyarthritis of the finger joints, hyperactive bowel sounds, and diffuse abdominal tenderness. Laboratory tests showed anemia (hemoglobin 6.3 g/dL, normal 12.1–15.1 g/dL), hypoalbuminemia (2.6 g/dL, normal 3.5–5.0 g/dL), elevated inflammatory markers (Erythrocyte sedimentation rate 120 mm/h, C‐reactive protein 3.3 mg/dL, normal 0–15 mm/h and <0.3 mg/dL, respectively), and elevated fecal calprotectin (>500 µg/g, normal 0–50 µg/g). A computed tomography (CT) scan of the abdomen and pelvis revealed findings suggestive of CD, with abnormal narrowing in the descending colon (Figure 1), and an inflammatory stricture and partial obstruction of the distal ileum (Figure 2). Endoscopy and colonoscopy revealed a sigmoid ulcer; however, the procedure could not proceed beyond the sigmoid colon due to colonic stricture. Initial PARIS classification was A1b, L3, B2. Biopsies from the edge of the sigmoid ulcer showed granulation tissue with acute and chronic inflammation, and a cytomegalovirus (CMV) immunohistochemical stain was positive.

The patient was initially treated for CMV with a 2‐week course of oral acyclovir. However, a repeat colonoscopy after completing treatment revealed persistent ulceration and unsuccessful dilation of the colonic stricture, with biopsies negative for CMV staining. CMV copies remained undetectable by polymerase chain reaction both before and after treatment, yet there was no clinical improvement. This prompted the initiation of infliximab at conventional dosing of 5 mg/kg. Despite this, the patient's condition deteriorated after the second induction dose, manifesting as a left lower quadrant mass and symptoms of intestinal obstruction. An abdominal X‐ray confirmed the obstruction, showing dilated small bowel loops, along with bilious emesis and abdominal distention.

**Figure 3:**
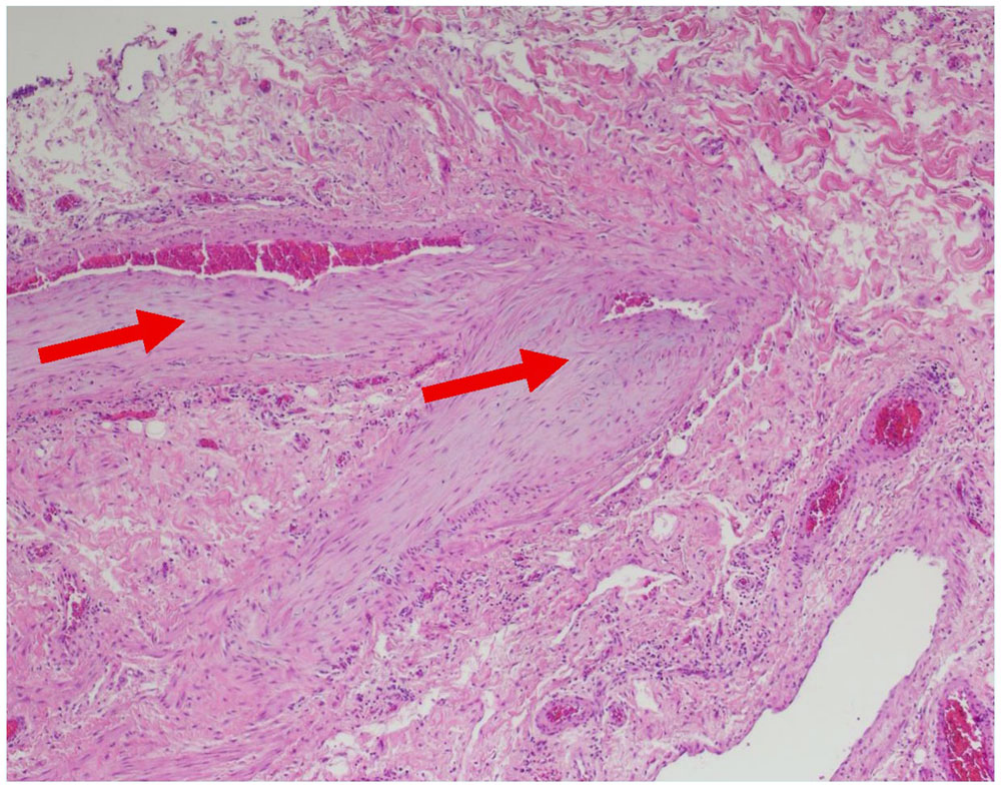
Arrows showing subintimal hyperplasia of medium‐sized arteries within the submucosa (Hematoxylin and eosin/×4).

Consequently, an exploratory laparotomy was performed, which included small bowel resection and the creation of an ileostomy with a mucous fistula. Pathology of the distal ileum showed subserosal lesions with lymphohistiocytic vasculitis (Figure 3) and eosinophil‐rich granulomas, consistent with eosinophilic vasculitis (Figure S1). She responded well to high‐dose steroids, six doses of intravenous cyclophosphamide, and transitioned to azathioprine with a starting dose of 1.5 mg/kg/day as recommended for esinophilic vasculitis, with continued drug level monitoring.^5^ After 9 months, the ileostomy was reversed, and she was in remission upon transition to adult gastroenterology.

## 3 DISCUSSION

EMV is a rare and specific type of intestinal vasculitis, which encompasses a spectrum of conditions involving inflammation of gastrointestinal blood vessels. While EMV primarily features eosinophilic infiltration and granuloma formation, other forms of intestinal vasculitis may differ in histological or clinical presentation.^4^

Eosinophils are involved in various immune responses and have been implicated in several systemic inflammatory disorders, including vasculitis. EMV is a rare condition that can affect small, medium, or large blood vessels, including those in the gastrointestinal tract. The pathophysiology of EMV is not fully understood but involves interleukins and eotaxins in eosinophil activation and inflammation.^6^ EMV can present with abdominal pain, gastrointestinal bleeding, and potential perforation, mimicking IBD. Additionally, elevated inflammatory markers, presence of strictures, and the overlapping extraintestinal manifestations contribute to the diagnostic challenge. While both EMV and CD respond to steroids, EMV is less likely to respond to standard IBD therapies. Diagnosis requires tissue pathology, including eosinophilic infiltration, necrotizing vasculitis, and extravascular granuloma formation, which may not be apparent in early stages.^4^

Treatment for EMV primarily involves glucocorticoids, with additional immunosuppressants such as cyclophosphamide, methotrexate, azathioprine, and cyclosporine for induction and maintenance. Biologics may be used in refractory cases.^5^

The presence of eosinophils on histopathology in patients with IBD is commonly observed. Studies suggest that eosinophils could be involved in the destructive inflammatory process, but also in tissue repair and wound healing. Their presence has been associated with more aggressive disease process and can mimic other diseases such as eosinophilic colitis, parasitic infections, allergic reactions, hypereosinophilic syndrome, and EMV.^7^

EMV and CD have different mechanisms and can involve different organs, yet both can present with extraintestinal manifestations (EIM). Differentiating between them in the early stages can be challenging. EMV EIM results from eosinophilic mediated damage to blood vessels in other organs such as heart and lungs, while EIM in CD is a T‐cell mediated inflammation that commonly affects skin, joints, and hepatobiliary system. Further, colonic strictures are less common than terminal ileal strictures in CD.^6^

A comparison of the distinguishing features of CD and EMV is provided in Table S1.

The 1990 American College of Rheumatology criteria and the 1994 Chapel Hill Consensus Conference definitions provide diagnostic frameworks for systemic vasculitis. While the patient's presentation with eosinophilic mesenteric vasculitis and autoimmune hemolytic anemia overlaps with features of systemic vasculitis, it does not fully meet the criteria for conditions without the involvement of other organs beyond the gastrointestinal tract and AHA. This highlights the need for broader consideration of vasculitis syndromes in patients presenting with eosinophilic inflammation and autoimmune phenomena.^8^

## 4 CONCLUSION

Eosinophilic mesenteric vasculitis is a rare form of intestinal vasculitis that can mimic IBD. The diagnosis is challenging and should be considered in the differential diagnosis for patients presenting with abdominal pain and gastrointestinal bleeding. While both IBD and EMV respond to steroids and immune suppression, EMV may involve a higher risk of surgical intervention.

## CONFLICT OF INTEREST STATEMENT

The authors declare no conflict of interest.

## ETHICS STATEMENT

Consent was obtained from the patient and parent.

## Supporting information

Supporting information.

Supporting information.
