# Supplementary figures and images for: Eosinophilic mesenteric vasculitis presenting as inflammatory bowel disease
Source: JPGN Rep. 2025 May 20;6(3):316–9. doi: 10.1002/jpr3.70035 (PMC12350026; doi:10.1002/jpr3.70035)

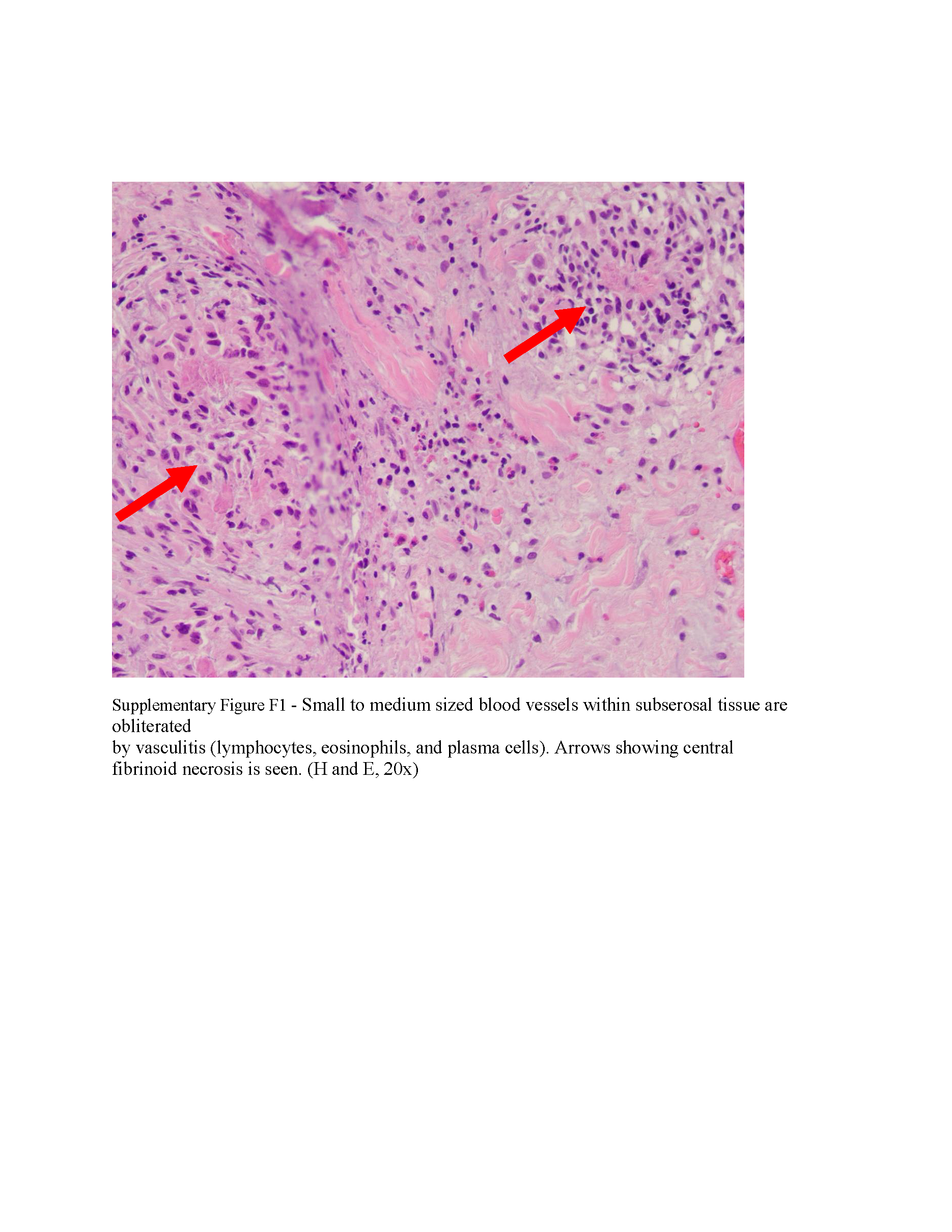

Supplement: Supplementary file 1 — Supporting information. [file JPR3-6-316-s001.tiff]
