# Supplementary material for: Eosinophilic mesenteric vasculitis presenting as inflammatory bowel disease
Source: JPGN Rep. 2025 May 20;6(3):316–9. doi: 10.1002/jpr3.70035 (PMC12350026; doi:10.1002/jpr3.70035)
Supplement: Supplementary file 2 — Supporting information. [file JPR3-6-316-s002.docx]

| Feature | Crohn's Disease (CD) | Eosinophilic Mesenteric Vasculitis (EMV) |
| --- | --- | --- |
| Pathophysiology | T-cell-mediated inflammation affecting entire GI tract | Eosinophilic-mediated inflammation of mesenteric vessels |
| Affected Regions | Anywhere in the GI tract; terminal ileum common | Primarily mesentery and associated vessels |
| Histological Findings | Non-caseating granulomas, transmural inflammation | Eosinophilic infiltration, necrotizing vasculitis, granulomas |
| Clinical Presentation | Abdominal pain, diarrhea, weight loss, growth failure | Abdominal pain, weight loss, joint pain, potential bowel obstruction |
| Extraintestinal Manifestations (EIM) | Skin, joints, eyes, hepatobiliary system (T-cell mediated) | Heart, lungs, and other organs (eosinophilic damage) |
| Response to Steroids | Typically responsive | Responsive |
| Response to Biologics | Effective (e.g., infliximab, adalimumab) | Typically ineffective |
| Imaging Findings | Bowel wall thickening, skip lesions, strictures | Mesenteric thickening, isolated strictures |
| Laboratory Findings | Elevated CRP, ESR, fecal calprotectin, anemia | Elevated eosinophil count, CRP, ESR |
| Treatment | Steroids, biologics, immunosuppressants, surgery if needed | Steroids, immunosuppressants (e.g., cyclophosphamide), surgery for complications |

**Supplemental Table 1: Comparison of Crohn's Disease (CD) and Eosinophilic Mesenteric Vasculitis (EMV)**

*GI: Gastrointestinal, CRP: C-reactive protein, ESR: Erythrocyte sedimentation rate, EIM: Extraintestinal manifestations*
